# High Levels of Nucleolar Spindle-Associated Protein and Reduced Levels of BRCA1 Expression Predict Poor Prognosis in Triple-Negative Breast Cancer

**DOI:** 10.1371/journal.pone.0140572

**Published:** 2015-10-20

**Authors:** Li Chen, Liu Yang, Feng Qiao, Xin Hu, Shan Li, Ling Yao, Xue-Li Yang, Zhi-Ming Shao

**Affiliations:** 1 Department of Breast Surgery, Fudan University Shanghai Cancer Center, Fudan University, Shanghai, China; 2 Department of Oncology, Shanghai Medical College, Fudan University, Shanghai 200032, China; 3 Department of Cancer Stem Cell, Dalian Medical University, Dalian, China; INRS, CANADA

## Abstract

**Purpose:**

Nucleolar spindle-associated protein (NuSAP1) is an important mitosis-related protein, and aberrant NuSAP1 expression is associated with abnormal spindles and mitosis. This study investigated the prognostic value of NuSAP1 in breast cancer.

**Methods:**

Two sets of tissue microarrays (TMAs) that included samples from 450 breast cancer patients were constructed, of which 250 patients were training set and the other 200 patients were validation set. Immunohistochemical staining was performed to determine the NuSAP1 levels. A Kaplan-Meier analysis was used to estimate the prognostic value of NuSAP1 in breast cancer. A stepwise Cox analysis was performed to construct a risk-prediction model for triple-negative breast cancer (TNBC). All statistical analysis was performed with SPSS software.

**Results:**

There were 108 (43.5%) and 88 (44.0%) patients expressed NuSAP1 in the training set and validation set respectively. High levels of NuSAP1 expression were related to poor disease-free survival (DFS) in both training (*P* = 0.028) and validation (*P* = 0.006) cohorts, particularly in TNBC. With combination of two cohorts, both NuSAP1 (HR = 4.136, 95% CI: 1.956–8.747, *P* < 0.001) and BRCA1 (HR = 0.383, 95% CI: 0.160–0.915, *P* = 0.031) were independent prognostic indicators of DFS in TNBC. A receiver operating characteristic (ROC) analysis revealed that the combination of NuSAP1 and BRCA1 significantly improved the prognostic power compared with the traditional model (0.778 versus 0.612, P < 0.001).

**Conclusions:**

Our study confirms the prognostic value of NuSAP1 in breast cancer. The combination of NuSAP1 and BRCA1 could improve the DFS prediction accuracy in TNBC.

## Introduction

Breast cancer is the most common type of cancer in women worldwide, and approximately 1.2 million new cases and 465,000 deaths occur each year[1, 2]. Therefore, breast cancer is one of the most serious health problems for women. Early diagnosis and timely treatment are the most effective strategies for fighting breast cancer. However, an effective marker for breast cancer diagnosis or prognosis has not yet been identified. Increasing amounts of evidence indicate that cancers are often heterogeneous and that the response to treatment depends on the subtype of breast cancer[3, 4]. Treatment with the guidance of molecular subtypes is important. Triple-negative breast cancer (TNBC) is a subtype of breast cancer with estrogen receptor (ER) negative, prognostic receptor (PR) negative, and human epidermal growth factor receptor 2 (HER-2) negative. BRCA1 is responsible for DNA repair and has been closely related to breast cancer, particularly TNBC[5–7]. More recently, the androgen receptor (AR) has been identified as a new marker of a specific subtype of TNBC[8–10]. However, with high heterogeneity, treatment of TNBC has always been a challenge. Therefore, additional efforts should be expanded to identify new indicators of breast cancer prognosis, especially for TNBC.

During mitosis, accurate cell division is required for the generation of two genetically identical daughter cells. The entire process must be performed with high fidelity to ensure that the duplicated chromosomes are equally distributed, and this process requires the coordinated operation of numerous proteins. Nucleolar-spindle associated protein (NuSAP1) is a microtubule- and chromatin-binding protein that stabilizes microtubules to prevent depolymerization, maintains spindle integrity, and further cross-links spindles into aster-like structures, fibers and networks[11–14]. NuSAP1 is transported into the nucleolus by importins and localizes to the chromatin-proximal microtubules throughout metaphase and anaphase. NuSAP1 is essential for mitosis from the stages of spindle assembly to cytokinesis. The overexpression of NuSAP1 results in the profound bundling of spindle microtubules. In contrast, the depletion of NuSAP1 by RNA interference results in G2-M arrest, aberrant mitotic spindles, cytokinesis, reductions in spindle microtubules, and abnormal chromosome segregation. Consequently, the aberrant expression of NuSAP1 has been associated with defective embryogenesis and cancer.

NuSAP1 is overexpressed and related to poor prognosis in hepatic carcinomas[15]. NuSAP1 has also been related to lung adenocarcinoma, cervical cancer, melanoma, meningioma, pituitary adenoma, and prostate cancer[16–20]. In the setting of breast cancer, Dilek Colak et al. reported that NuSAP1 expression significantly differs between ductal carcinoma in situ (DCIS) and invasive ductal carcinoma (IDC)[21]. Therefore, NuSAP1 might be involved in tumorigenesis and progression. However, the NuSAP1 expression status of the subtypes of breast cancer remains unknown. The current study investigated the correlation between NuSAP1 expression and the prognosis of different subtypes of breast cancer, particularly TNBC.

## Materials and Methods

### Ethics Statement

The study has been approved by the Human Research Ethics Committee of Fudan University Shanghai Cancer Center, Shanghai, China. The approved number of ethics committee is 050432–4. Written informed consent was provided by all patients. All samples and medical data used in this study have been anonymized.

### Patients and specimens

The present study included 450 patients who were diagnosed with stage I to III primary breast cancer from August 2001 to March 2006 according to histopathological analysis conducted at Fudan University Shanghai Cancer Center (FDUSCC). There were a training set and a validation set with 250 and 200 patients, respectively. All patients were subjected to either a mastectomy and axillary lymph node dissection or breast conservation surgery. The clinicopathological information, including age, menopausal status, tumor size, lymph node status, grade, ER, progesterone receptor (PR), HER-2 status, and TNM stage, was collected and shown in Table 1. Patients were further classified into four subtypes according St Gallen International Breast Cancer Conference (2011) Expert Panel[22]. All patients were regularly followed, and the median follow-up time was 96 months.

**Table 1:** Correlation between clinicopathologic variables and expression of NuSAP1.

	Training set				Validation set			
Variables	Number of patients	NuSAP1 expression		*P* ^a^ value	Number of patients	NuSAP1 expression		*P* ^a^ value
		Negative n (%)	Positive n (%)			Negative n (%)	Positive n (%)	
Total	248	140(56.5)	108(43.5)		200	112(56.0)	88(44.0)	
Age				0.275				0.053
≤ 50	120	72(29.0)	48(19.3)		113	70(35.0)	43(21.5)	
> 50	128	68(27.5)	60(24.2)		87	42(21.0)	45(22.5)	
Menopausal status				0.611				0.944
Premenopause	108	59(23.8)	49(19.8)		121	68(34.0)	53(26.5)	
Postmenopause	137	81(32.7)	59(23.7)		79	44(22.0)	35(17.5)	
Tumor size				0.529				0.285
≤ 2cm	115	66(26.2)	50(20.2)		101	51(25.5)	50(25.0)	
> 2, ≤ 5cm	119	65(26.2)	54(21.8)		91	56(28.0)	35(17.5)	
> 5cm	14	10(4.1)	4(1.5)		8	5(2.5)	3(1.5)	
Lymph node status				0.949				0.682
Negative	151	85(34.3)	66(26.6)		99	54(27.0)	45(22.5)	
Positive	97	55(22.2)	42(16.9)		101	58(29.0)	43(21.5)	
Grade				0.627				0.158
1	5	3(1.2)	2(0.8)		2	0(0.0)	2(1.0)	
2	183	100(40.4)	83(33.5)		142	77(38.5)	65(32.5)	
3	60	37(14.9)	23(9.2)		56	35(17.5)	21(10.5)	
ER status				0.741				0.936
Negative	143	82(33.1)	61(24.6)		112	63(31.5)	49(24.5)	
Positive	105	58(23.4)	47(18.9)		88	49(24.5)	39(19.5)	
PR status				0.710				0.434
Negative	185	106(42.7)	79(31.9)		113	66(33.0)	47(23.5)	
Positive	63	34(13.8)	29(11.6)		87	46(23.0)	41(20.5)	
HER-2 status				0.522				0.393
Negative	148	86(34.7)	62(25.0)		100	53(26.5)	47(23.5)	
Positive	100	54(21.8)	46(18.5)		100	59(29.5)	41(20.5)	
BRCA1 (IHC)				0.006				0.006
Negative	128	83(33.5)	45(18.1)		106	69(34.5)	37(18.5)	
Positive	120	57(23.0)	63(25.4)		94	43(21.5)	51(25.5)	
TNM				0.924				0.514
I	74	42(16.9)	32(12.9)		65	33(16.5)	32(16.0)	
II	133	76(30.5)	57(23.0)		126	73(36.5)	53(26.5)	
III	41	22(8.9)	19(7.6)		9	6(3.0)	3(1.5)	
Subtype				0.063				0.169
Luminal A	48	29(11.7)	19(7.7)		50	29(14.5)	21(10.5)	
Luminal B	57	29(11.7)	28(11.3)		50	25(12.5)	25(12.5)	
Her-2 overexpression	43	25(10.1)	18(7.2)		50	34(17.0)	16(8.0)	
Triple-negative	100	57(23.0)	43(17.3)		50	24(12.0)	26(13.0)	

**Abbreviations**: NuSAP1, Nucleolar spindle-associated protein; ER, estrogen receptor; PR, progesterone receptor; HER-2, human epidermal growth factor receptor 2; BRCA1,breast cancer type 1 susceptibility protein.

*P* ^a^ value was calculated using Pearson's χ.

### Breast cancer tissue microarray construction

The breast cancer tissue samples used to construct the tissue microarrays (TMAs) were obtained before treatment, fixed in formalin and embedded in paraffin. Tumor regions were stained with hematoxylin and eosin (HE) to identify representative tumor regions from which two 1.0-mm tissue cores were retrieved and transferred into recipient array blocks using a tissue micro arrayer (UNITMA Instruments, Seoul, Korea). TMAs were composed of duplicate cores from different areas of the same tumor to compare staining patterns in our research. Two sets of TMAs were generated by the Department of Pathology of FDUSCC with 250 patients and 200 patients, respectively.

### Immunohistochemistry

The TMAs were subjected to immunohistochemical staining for the NuSAP1 and BRCA1 proteins with a 2-step protocol (GTVisionTMIII). NuSAP1 was detected with a rabbit anti-NuSAP1 polyclonal antibody (Proteintech Group, Chicago, IL, USA), and BRCA1 was detected with mouse anti-BRCA1 (Santa Cruz Biotechnology, Dallas, Texas, USA). The TMAs were deparaffinized with xylene, gradually rehydrated in a gradient ethanol series and then rinsed with phosphate-buffered saline (PBS) prior to NuSAP1 or BRCA1 immunohistochemical staining. Antigen retrieval was performed by immersing the sections in 0.01 M Tris–sodium citrate (pH 6.0). After boiling at 121°C for 10 min, the sections were incubated with NuSAP1 or BRCA1 for 2 minutes. After blocking for 20 minutes, the slides were subjected to anti-NuSAP1 (1:200) or anti-BRCA1 (1:200) primary antibodies at 4°C overnight. HRP-conjugated secondary antibodies were used to detect the primary antibodies with subsequent colorimetric detection using 3, 3-diaminobenzidine (DAB). The TMAs were then counterstained with Gill hematoxylin and dehydrated in an ascending ethanol series before being cleared with xylene and mounted with a coverslip.

### Evaluation of the immunostaining for NuSAP1 and BRCA1

For each antibody, the TMAs were stained and semi-quantitatively scored according to a staining index (SI; range 0–9) with the following formula: SI = intensity × proportion scores. The staining intensities were classified into three grades (1: weak, 2: moderate, and 3: strong), and proportion scores were assigned based on the percentages of stained cells (0:0%, 1: < 10%, 2: 10–50%, and 3: 50–100%). For NuSAP1 and BRCA1, SIs ≥ 5 were considered positive staining, whereas SIs < 5 were defined as negative staining. Two experienced pathologists who were blinded to all clinical data conducted the scoring in parallel.

### Statistical analysis

The associations between the clinicopathological parameters and NuSAP1 expression were evaluated with Pearson χ2, and Fisher’s exact tests as appropriate. A Kaplan-Meier (KM) analysis and log-rank test were performed to determine the correlation between NuSAP1 expression and disease-free survival (DFS) and overall survival (OS). Univariate and multivariate analysis of the DFS were performed with Cox risk proportion models. *P* < 0.05 was considered to indicate significant differences. The statistical analysis was performed using SPSS (version 13.0; SPSS, Chicago, IL, USA).

## Results

### Clinicopathological characteristics and NuSAP1 expression in breast cancer patients

**Fig 1:**
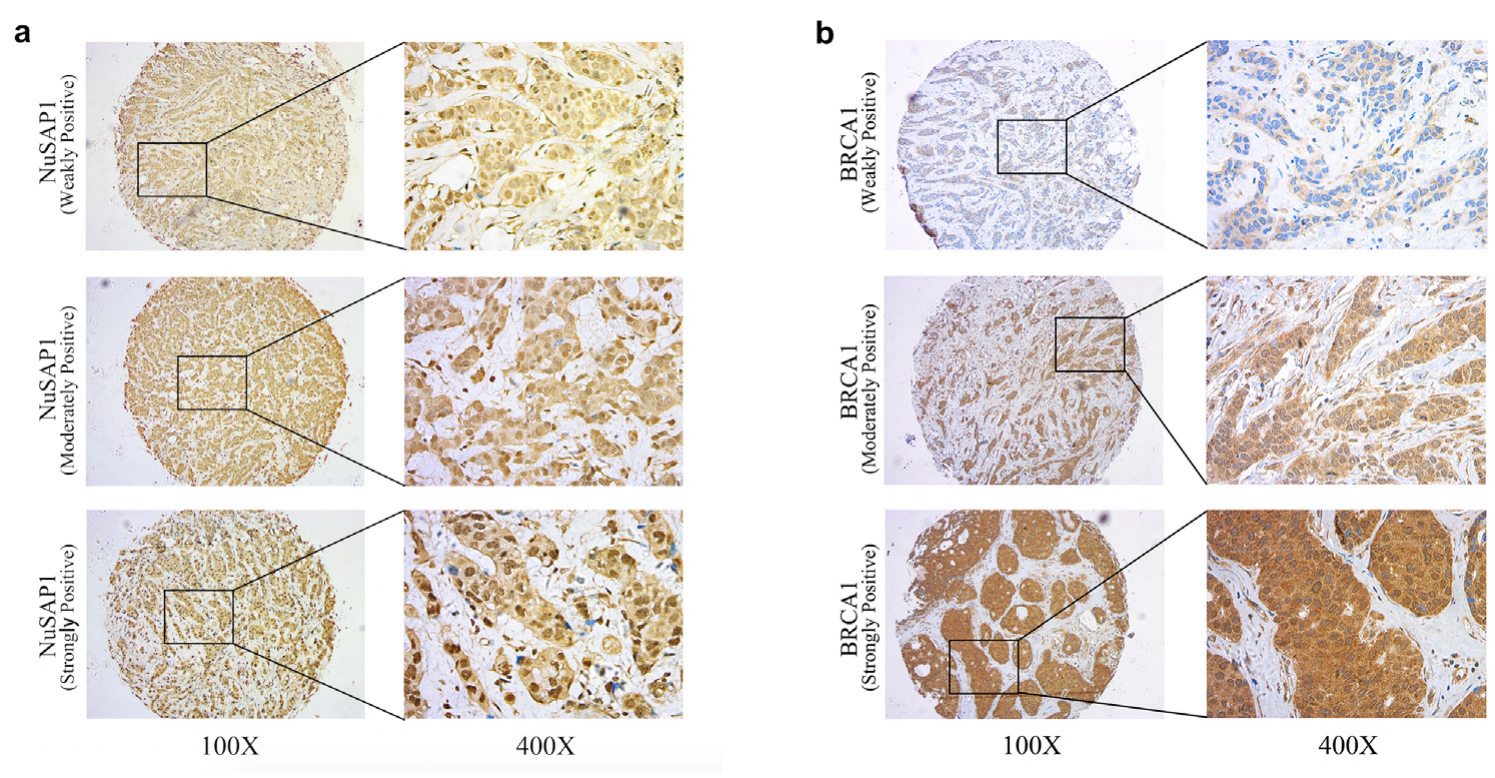
Representative NuSAP1 (a) and BRCA1 (b) immunohistochemical staining of malignant cells from breast cancer tissue specimens at low magnification (100X) and high magnification (400X). The staining intensities were classified as weak (score of 1), moderate (score of 2), or strong (score of 3).

In training set, a total of 250 female breast cancer samples were collected, but two of the samples lacked follow-up data. Thus, the remaining 248 samples were included in the subsequent analysis. All patients were female and had been diagnosed with stages I to III primary breast cancer at a median age of 51 years. The ER, PR, and HER-2 statuses were collected, and the patients were classified into four subtypes, i.e., Luminal A, Luminal B, HER-2 overexpression, and TNBC. The ER, PR and HER-2 subtypes were defined based on immunohistochemistry (IHC) and fluorescence in situ hybridization (FISH) results. Of the patients, 19.4%, 23.0%, 17.3% and 40.3% were classified as Luminal A, Luminal B, HER-2 overexpression, and TNBC subtypes, respectively. To investigate the clinical function of NuSAP1 in breast cancer, its expression in the cohort was examined by immunohistochemistry (Fig 1a). As shown in Table 1, 108 (43.5%) of the samples expressed NuSAP1 protein and 140 (56.5%) samples did not in training set. Specifically, among the 108 NuSAP1-positive patients, the Luminal A, Luminal B, HER-2 overexpression and Triple-negative subgroups included 19 (7.7%), 28 (11.3%), 18 (7.2) and 43 (17.3) patients, respectively. NuSAP1 expression was not related to ER, PR or HER-2 status (Table 1). However, the association between the expression of NuSAP1 and breast cancer subtypes bordered on significant (*P* = 0.063). As early as 1995, Marilyn E. Thompson et al. reported that BRCA1 expression decreases during the progression of breast cancer[23]. BRCA1 has long been known to be associated with breast cancer and ovarian cancer[24]. Interestingly, the expression levels of NuSAP1 and BRCA1 were significantly correlated in our patient cohort (*P* = 0.006). Similar correlation was found in validation set (Table 1, P = 0.006).

### Univariate and multivariate analysis of breast cancer

Univariate analysis was performed to evaluate the correlations between clinicopathological parameters and DFS, and several factors were significantly associated. As shown in Table 2, in training set, tumor size > 5 cm, positive lymph node status, grade 3 status, and positive NuSAP1 expression were associated with a greater risk of recurrence and a lower DFS (*P* = 0.016). In contrast, PR expression was related to improved DFS for all patients (*P* = 0.033). Accordingly, a stepwise multivariate analysis that included age, menopausal status, lymph node status, PR, and NuSAP1 expression was conducted. Menopausal status, lymph node status, PR, and NuSAP1 were identified as significant prognostic factors for DFS (Table 3, *P* < 0.05). In validation set, we found NuSAP1 was significantly prognostic for DFS (Tables 2 and 3, *P* < 0.05).

**Table 2:** Univariate analysis for disease-free survival.

Variables	Training set		Validation set	
	HR (95% CI)	*P* ^a^ value	HR (95% CI)	*P* ^a^ value
Age				
≤50 years	1		1	
>50years	0.965(0.566–1.647)	0.897	0.873(0.463–1.645)	0.674
Menopausal status				
Premenopause	1		1	
Postmenopause	1.582(0.905–2.767)	0.108	1.333(0.708–2.512)	0.374
Tumor size				
≤2cm	1		1	
>2, 5≤cm	1.338(0.752–2.378)	0.322	1.002(0.134–7.504)	0.998
>5cm	4.817(2.030–11.429)	0.000	1.182(0.158–8.845)	0.870
Lymph node status				
Negative	1		1	
Positive	2.175(1.273–3.716)	0.004	1.400(0.742–2.640)	0.298
Grade				
1 or 2	1		1	
3	1.756(1.004–3.071)	0.048	1.098(0.535–2.255)	0.799
ER status				
Negative	1		1	
Positive	0.836(0.483–1.446)	0.522	0.535(0.275–1.043)	0.066
PR status				
Negative	1		1	
Positive	0.421(0.190–5.0.933)	0.033	0.695(0.361–1.338)	0.276
HER-2 status				
Negative	1		1	
Positive	1.013(0.590–1.737)	0.964	1.493(0.775–2.874)	0.230
NuSAP1				
Negative	1		1	
Positive	1.948(1.132–3.354)	0.016	2.458(1.272–4.750)	0.007

**Abbreviations**: NuSAP1, Nucleolar spindle-associated protein; ER, estrogen receptor; PR, progesterone receptor; HER-2, human epidermal growth factor receptor 2; BRCA1, breast cancer type 1 susceptibility protein.

*P* ^a^ value was calculated using Pearson's χ.

**Table 3:** Multivariate analysis for disease-free survival.

Variables	Training set		Validation set	
	HR (95% CI)	*P* ^a^ value	HR (95% CI)	*P* ^a^ value
Age	0.586(0.304–1.128)	0.110	0.835(0.371–1.877)	0.662
Menopausal status	2.010(1.011–3.995)	0.046	1.464(0.648–3.308)	0.359
Lymph node status	2.232(1.298–3.837)	0.004	1.553(0.802–3.005)	0.191
PR	0.380(0.171–0.846)	0.018	0.691(0.351–1.361)	0.285
NuSAP1	2.102(1.220–3.621)	0.007	2.606(1.338–5.076)	0.005

**Abbreviations**: NuSAP1, Nucleolar spindle-associated protein; PR, progesterone receptor.

*P* ^a^ value was calculated using Pearson's χ.

### NuSAP1 expression was associated with poor DFS in breast cancer, particularly in TNBC

**Fig 2:**
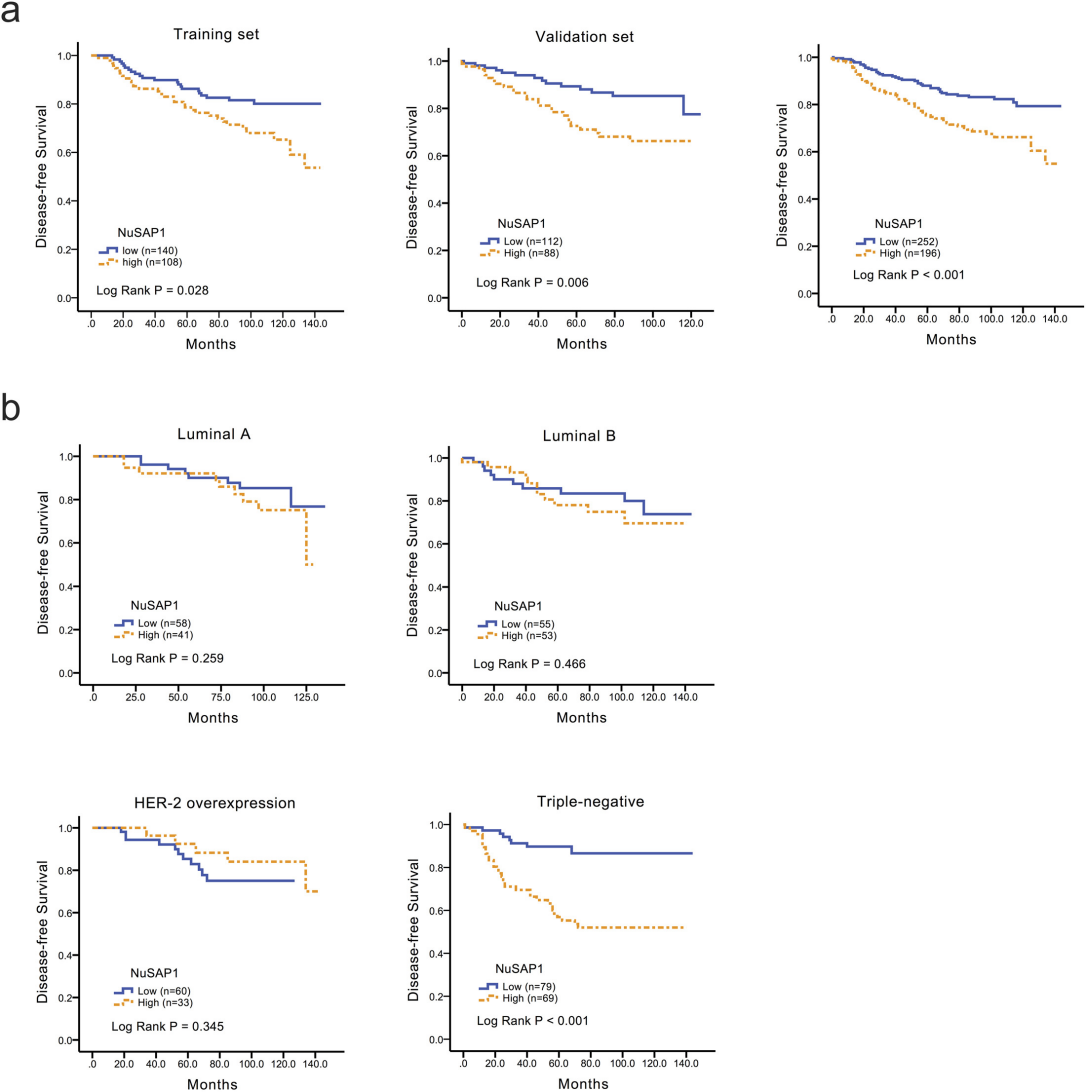
Low expression of NuSAP1 favored DFS, particularly for TNBC patients. Cumulative DFS curves for breast cancer patients classified as the total group (a) training set: NuSAP1+ (n = 108) and NuSAP1- (n = 140); validation set: NuSAP1+ (n = 88) and NuSAP1- (n = 112); overall: NuSAP1+ (n = 196) and NuSAP1- (n = 252); and (b) overall population luminal A, NuSAP1+ (n = 41) and NuSAP1- (n = 58); luminal B, NuSAP1+ (n = 53) and NuSAP1- (n = 55); HER-2 overexpression, NuSAP1+ (n = 33) and NuSAP1- (n = 60); triple-negative NuSAP1+ (n = 69) and NuSAP1- (n = 79) subgroups.

To explore the prognostic value of NuSAP1 for DFS and OS of the breast cancer patients, a KM analysis of all patients was performed. As shown in Fig 2a, the expression of NuSAP1 was generally associated with a poor DFS in both training (*P* = 0.028) and validation cohort (*P* = 0.006). In all patients with combination of two cohorts, similar trends were found with P < 0.001; further analysis of the prognostic value of NuSAP1 in four subtypes of breast cancer revealed that NuSAP1 expression was significantly correlated with poor DFS in triple-negative subgroup (Fig 2b, *P* < 0.001). However, non-significant differences were observed in the other three subgroups (Fig 2b).

### NuSAP1 and BRCA1 were associated with DFS in TNBC

**Fig 3:**
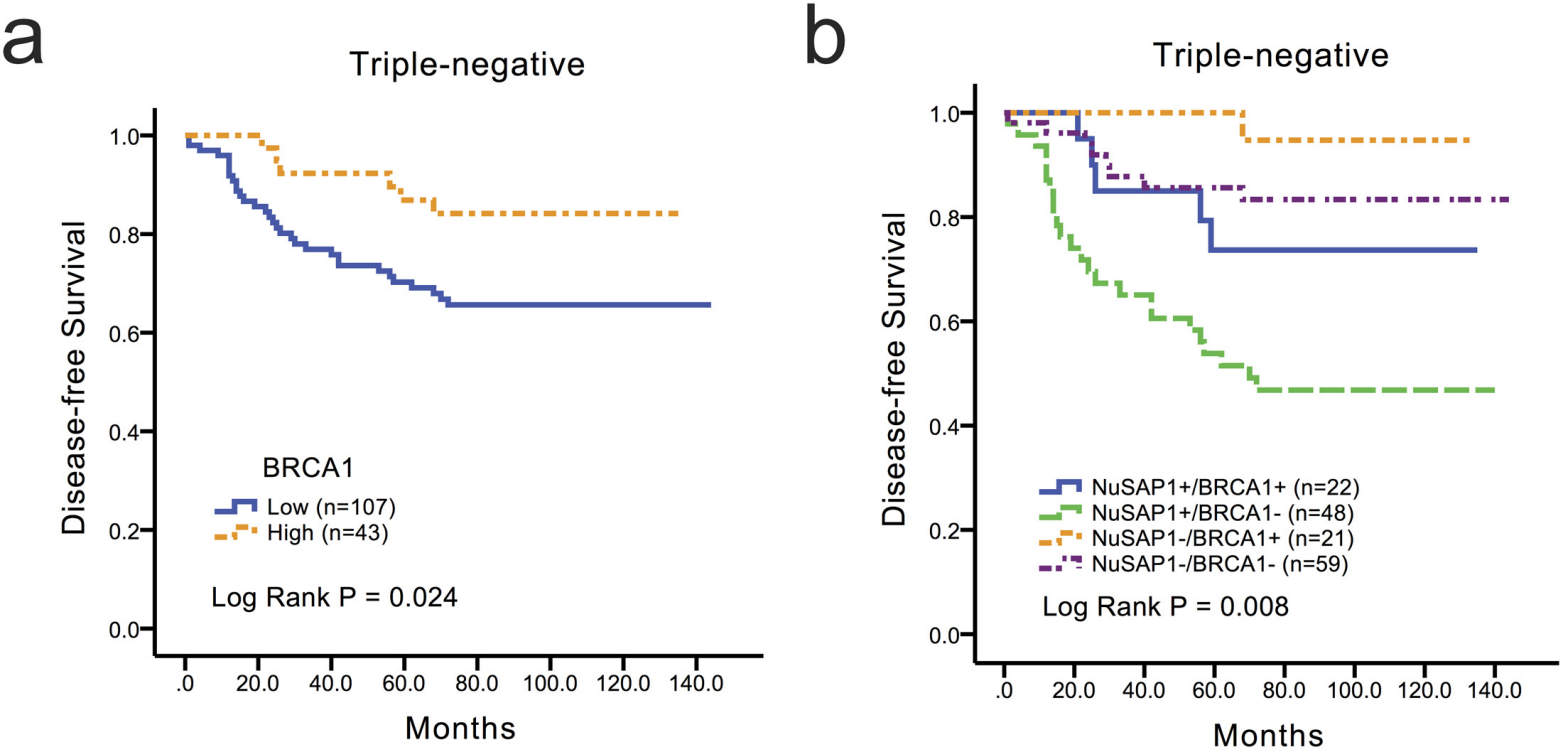
Low levels of NuSAP1 expression combined with high levels of BRCA1 expression favored DFS, particularly for TNBC patients. Cumulative DFS curves for the breast cancer classified as (a) the BRCA1+ (n = 43) and BRCA1- (n = 107) subgroup; and (b) the NuSAP1+/ BRCA1+ (n = 22); NuSAP1+/ BRCA1- (n = 48); NuSAP1-/ BRCA1+ (n = 21); and NuSAP1-/ BRCA1- (n = 59) subgroups.

In a study of various gene profiles that further classified TNBC into six subtypes with distinct characteristics, Brian D. Lehmann identified BRCA1 as an important molecular marker of TNBC; BRCA1 was included among the gene sets in their study[25]. In the current study, a KM analysis was performed to verify the prognostic values of BRCA1 in TNBC, and the expression of BRCA1 was related to improved DFS in all TNBC with combination of training and validation cohort patients (Fig 3a, *P* = 0.024). This finding was in agreement with that in general breast cancer[26]. Moreover, univariate and multivariate analysis were performed in the TNBC group (Tables 4 and 5). As shown in Table 4, tumor size > 5 cm, positive lymph node status, and positive NuSAP1 expression were significantly associated with worse DFS (*P* < 0.05). In contrast, positive BRCA1 expression was related to improved DFS (*P* = 0.031). Furthermore, the multivariate analysis found lymph nodes status, NuSAP1, and BRCA1 expression to be related to DFS in TNBC (Table 5).

**Table 4:** Univariate analysis for disease-free survival in TNBC.

	HR (95% CI)	*P* ^a^ value
Age		
≤50 years	1	
>50years	0.814(0.429–1.543)	0.528
Menopausal status		
Premenopause	1	
Postmenopause	1.771(0.923–3.395)	0.085
Tumor size		
≤2cm	1	
>2, ≤5cm	1.577(0.796–3.123)	0.192
>5cm	3.272(1.074–9.966)	0.037
Lymph node status		
Negative	1	
Positive	2.137(1.129–4.042)	0.020
Grade		
1 or 2	1	
3	1.035(0.535–2.001)	0.920
BRCA1		
Negative	1	
Positive	0.383(0.160–0.915)	0.031
NuSAP1		
Negative	1	
Positive	4.136(1.956–8.747)	0.000

**Abbreviations**: BRCA1, breast cancer type 1 susceptibility protein; NuSAP1, Nucleolar spindle-associated protein.

*P* ^a^ value was calculated using Pearson’s χ.

**Table 5:** Multivariate analysis for disease-free survival in TNBC.

	HR(95% CI)	*P* ^a^ value
Age	0.994(0.424–2.330)	0.989
Menopausal status	1.336(0.560–3.189)	0.514
Lymph node status	2.171(1.126–4.186)	0.021
BRCA1	0.390(0.162–0.940)	0.036
NuSAP1	4.388(2.048–9.400)	0.000

**Abbreviations**: BRCA1, breast cancer type 1 susceptibility protein; NuSAP1, Nucleolar spindle-associated protein.

*P* ^a^ value was calculated using Pearson’s χ.

### Prognostic value of the combined expression of NuSAP1 and BRCA1 for DFS in TNBC

Subsequently, we evaluated the combined predictive value of NuSAP1 and BRCA1 for DFS. All TNBC patients were classified into the following four subgroups: NuSAP1+/BRCA1+ (n = 22), NuSAP1+/BRCA1- (n = 48), NuSAP1-/BRCA1+ (n = 21), and NuSAP1-/BRCA1- (n = 59). As shown in Fig 3b, the NuSAP1+/BRCA1- patients exhibited worse DFS than the NuSAP1-/BRCA1+ (*P* = 0.008) subgroup.

### Predictive risk model of the combined expressions of NuSAP1 and BRCA1 for DFS in TNBC

**Fig 4:**
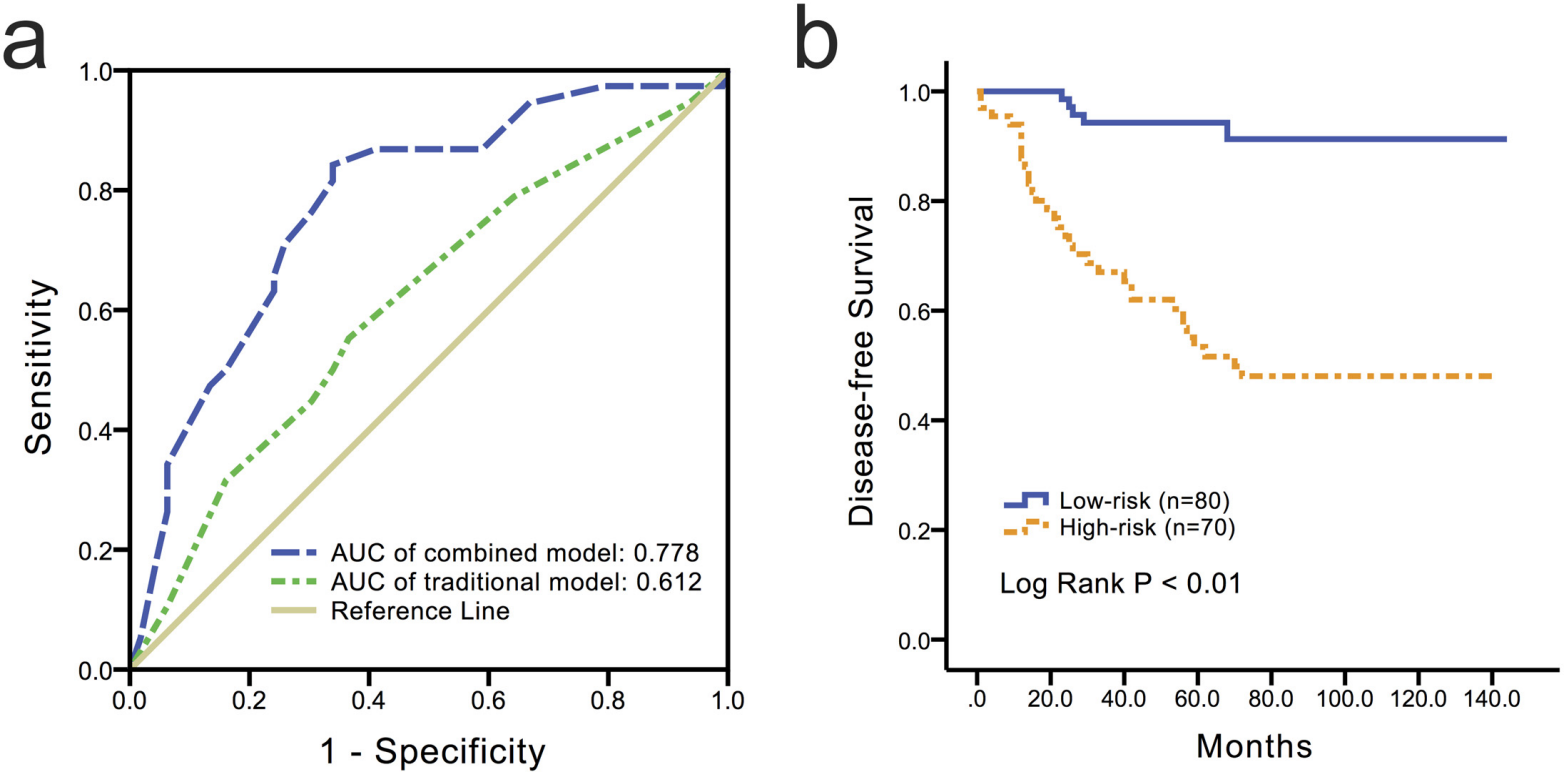
ROC curves showing that the combination of the NuSAP1 and BRCA1 expression levels (blue) improved the prognostic accuracy of the traditional model (green). All P < 0.01 in the AUC comparison.

Next, we sought to evaluate the capability of the combination of NuSAP1 and BRCA1 to identify the patients with TNBC who were more likely to experience DFS events. In the absence of NuSAP1 and BRCA1, the traditional model exhibited modest prognostic accuracy with a bootstrap-corrected AUC value of 0.612 (Fig 4a, 95% CI: 0.488–0.699). The addition of NuSAP1 and BRCA1 expression to the traditional model significantly improved the bootstrap-corrected AUC value to 0.778 (Fig 4a, 95% CI: 0.665–0.838). The traditional model (Mtraditional) was M_traditional_ = -0.025 * Age + 0.601 * Menopausal Status + 0.767 * Lymph Node Status.

The combined model (Mcombined) was M_combined_ = -0.006 * Age + 0.5290 * Menopausal Status + 0.775 * Lymph Node Status + 1.479 * NuSAP1–0.942 * BRCA1. The optimal cutoff value of the ROC curve was 0.821. The TNBC patient cohort was subsequently reclassified as high risk (risk score > 0.821, n = 80) or low risk (risk score ≤ 0.821, n = 70). The survival curves revealed a significant difference in survival between the two groups (Fig 4b, *P <* 0.01).

## Discussion

As a cell cycle-related protein, NuSAP1 plays a vital role in mitosis, and aberrant NuSAP1 expression results in abnormal mitotic spindles. NuSAP1 is upregulated and related to poor prognosis in many cancers. Dilek Colak et al. indicated that the NuSAP1 gene might be involved in the carcinogenesis and progression of breast cancer[21]. To further investigate the correlation between NuSAP1 expression and breast cancer prognosis, particularly for different subtypes, we constructed two sets of TMAs that contained 450 stage I to III primary breast cancer tissues and determined the NuSAP1 expressions via immunostaining. A KM plot was constructed to evaluate the prognostic value of NuSAP1, and high levels of NuSAP1 expression were found to be related with poor DFS for all patients. Specifically, this association was more significant in the triple-negative subgroup. No significant difference was observed between the Luminal A, Luminal B and HER-2 overexpression subgroups. Therefore, the association between NuSAP1 and DFS might have primarily derived from the triple-negative subgroup.

TNBC is a specific subtype of breast cancer that is negative for ER, PR and HER-2 expression. Due to the high level of heterogeneity and the lack of well-defined molecular targets, the treatment of TNBC has long been a challenge. Recently, Brian D. Lehmann et al. examined gene expression profiles and further divided TNBC into six subtypes that included two basal-like (BL) subtypes, an immunomodulatory subtype, a mesenchymal subtype, a mesenchymal stem-like subtype and a luminal androgen receptor subtype[25]. The basal-like subtypes included BL1 and BL2, both of which exhibited increased expressions of cell cycle and DNA damage response genes. Lehmann et al. also primarily observed BRCA1 enrichment in the BL1 subtype. In our study, high NuSAP1 expression levels indicated poor prognosis, which is consistent with the emerging role of NuSAP1 as a modulator of the relationship between the bundling of spindle microtubules and cancer[15, 18, 20]. In contrast, decreased BRCA1 expression indicated a poor prognosis in the TNBC group, as previously described[27]. In the current study, the DFS of TNBC patients could be stratified by the NuSAP1 and BRCA1 expression status. These findings indicated that the combination of these two molecular markers provided additional prognostic information. Thus, NuSAP1 might be a biomarker for TNBC.

The univariate and multivariate analysis demonstrated that NuSAP1 and BRCA1 were both independent prognostic factors of DFS in TNBC. Furthermore, a risk model that incorporated these two proteins could classify the TNBC patients into two recurrence risk categories. To the best of our knowledge, this study is the first to verify the prognostic value of the combination of NuSAP1 and BRCA1 in TNBC.

Our results are limited by the restricted sample size, particularly regarding TNBC. Therefore, our results should be validated in larger and consistent cohorts of breast cancer patients. TNBC were divided into six subgroups and subsequent investigations were needed to verify the specific type of NuSAP1 function.

In conclusion, our study confirmed the prognostic value of NuSAP1 in breast cancer. The combination of NuSAP1 and BRCA1 improved the DFS prediction accuracy in TNBC. Our findings may be used to advance the classification and treatment of specific breast cancer patients.

## Supporting Information

S1 Fig Kaplan-Meier estimates of the OS according to NuSAP1 expression. Cumulative OS curves for breast cancer patients (a) training set: NuSAP1+ (n = 108) and NuSAP1- (n = 140) patients; Validation set: NuSAP1+ (n = 88) and NuSAP1- (n = 112) patients; all patients: NuSAP1+ (n = 196) and NuSAP1- (n = 252) patients and (b) luminal A: NuSAP1+ (n = 41) and NuSAP1- (n = 58); luminal B: NuSAP1+ (n = 53) and NuSAP1- (n = 55); HER2-overexpression: NuSAP1+ (n = 33) and NuSAP1- (n = 60); triple-negative: NuSAP1+ (n = 69) and NuSAP1- (n = 79) patients.
(TIF)

S2 Fig Kaplan-Meier estimates of the OS according to BRCA1 expression in the TNBC subgroup. Cumulative DFS curves for TNBC patients classified as BRCA1+ (n = 43) and BRCA1- (n = 107).
(TIF)

S1 Table Correlations between NuSAP1 and BRCA1 expression in the TNBC subgroup. (DOCX)

S2 Table Correlations of the clinicopathologic variables with the expressions of BRCA1 in the TNBC subgroup. (DOCX)
