# Supplementary figures and images for: High Levels of Nucleolar Spindle-Associated Protein and Reduced Levels of BRCA1 Expression Predict Poor Prognosis in Triple-Negative Breast Cancer
Source: PLoS One. 2015 Oct 20;10(10):e0140572. doi: 10.1371/journal.pone.0140572 (PMC4618922; doi:10.1371/journal.pone.0140572)

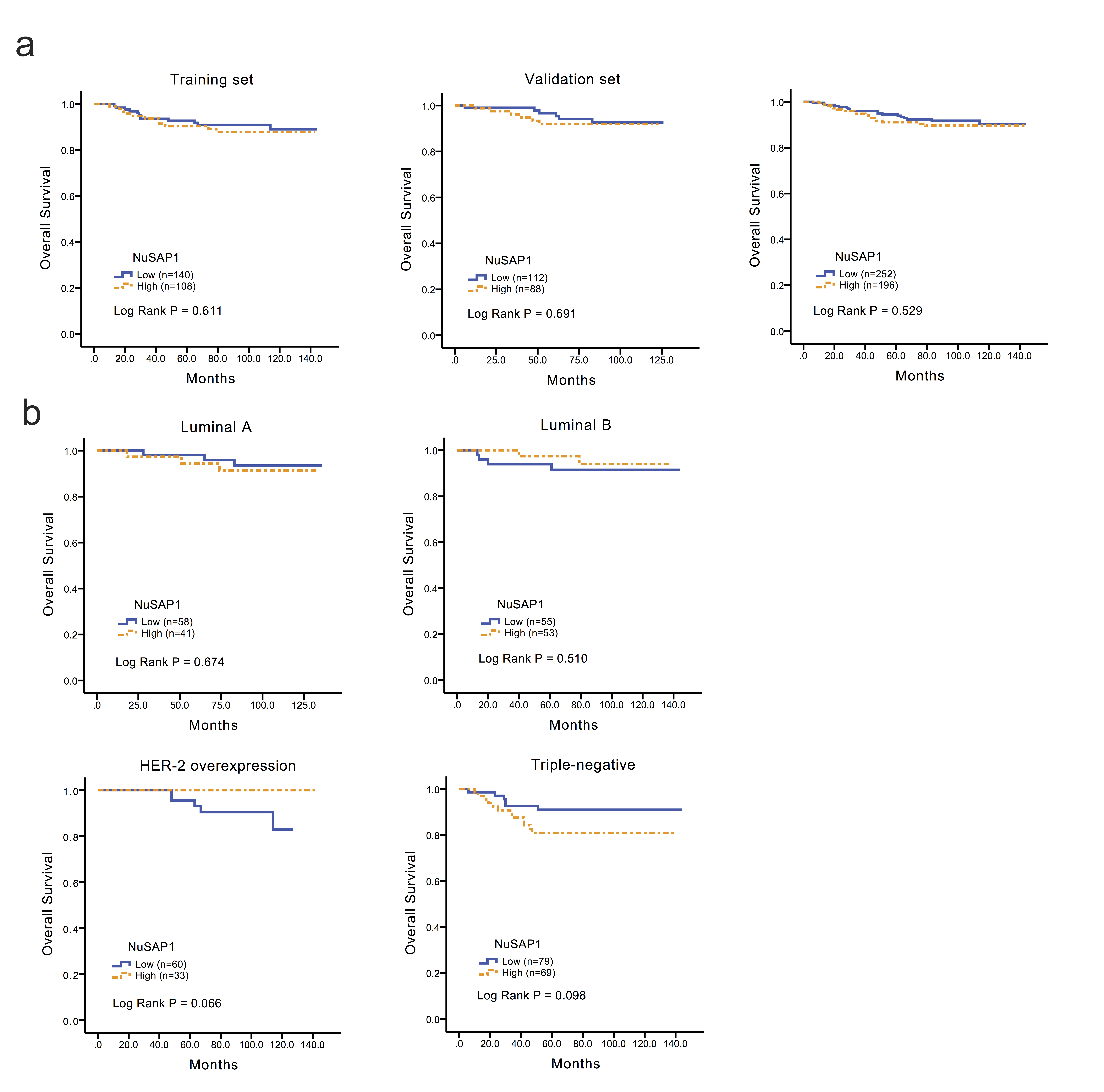

Supplement: S1 Fig — Cumulative OS curves for breast cancer patients (a) training set: NuSAP1+ (n = 108) and NuSAP1- (n = 140) patients; Validation set: NuSAP1+ (n = 88) and NuSAP1- (n = 112) patients; all patients: NuSAP1+ (n = 196) and NuSAP1- (n = 252) patients and (b) luminal A: NuSAP1+ (n = 41) and NuSAP1- (n = 58); luminal B: NuSAP1+ (n = 53) and NuSAP1- (n = 55); HER2-overexpression: NuSAP1+ (n = 33) and NuSAP1- (n = 60); triple-negative: NuSAP1+ (n = 69) and NuSAP1- (n = 79) patients. (TIF) [file pone.0140572.s001.tif]

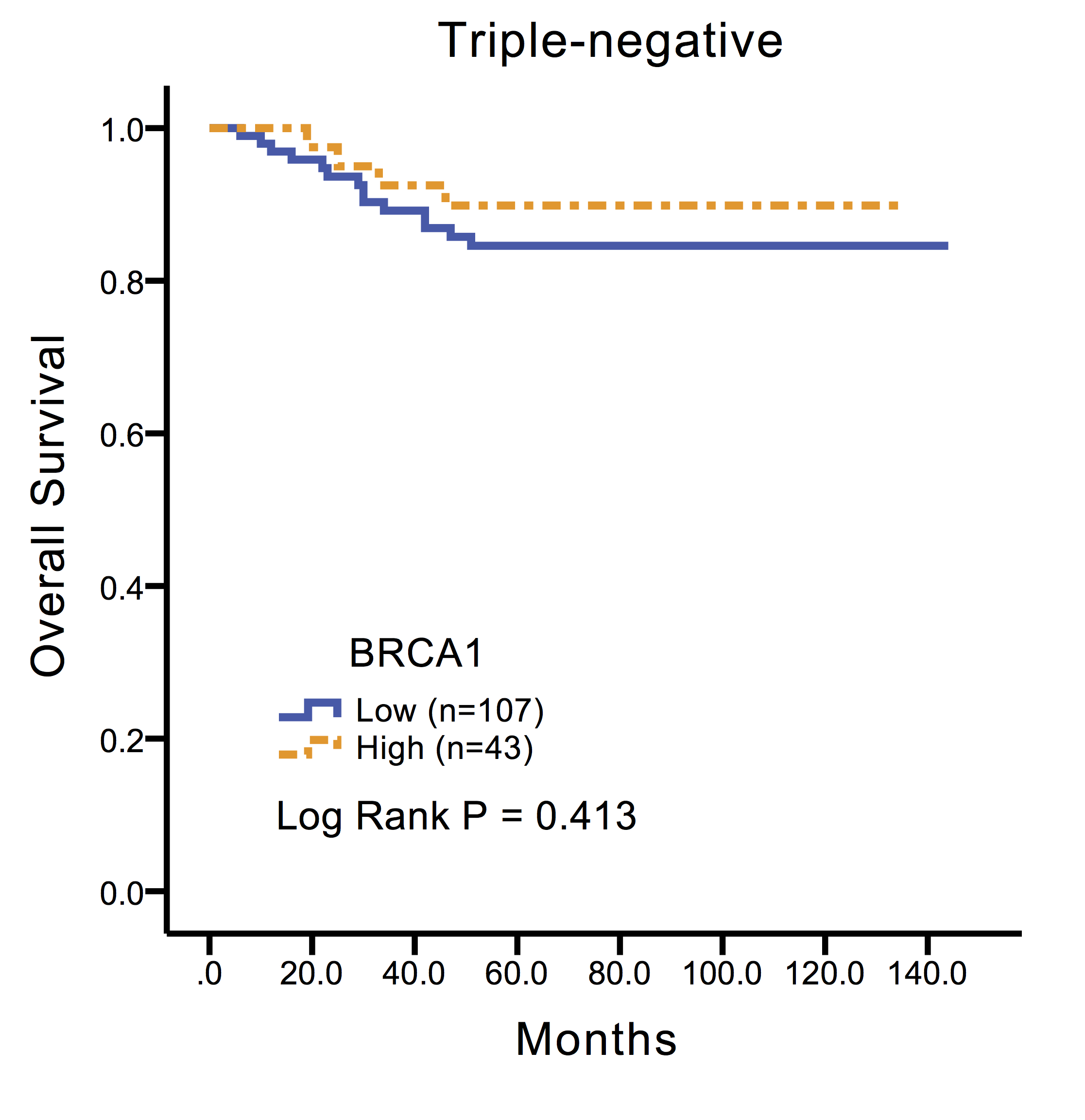

Supplement: S2 Fig — Cumulative DFS curves for TNBC patients classified as BRCA1+ (n = 43) and BRCA1- (n = 107). (TIF) [file pone.0140572.s002.tif]
