# Supplementary material for: High Levels of Nucleolar Spindle-Associated Protein and Reduced Levels of BRCA1 Expression Predict Poor Prognosis in Triple-Negative Breast Cancer
Source: PLoS One. 2015 Oct 20;10(10):e0140572. doi: 10.1371/journal.pone.0140572 (PMC4618922; doi:10.1371/journal.pone.0140572)
Supplement: S1 Table — (DOCX) [file pone.0140572.s003.docx]

| **Table S1** Correlation of the expression of NuSAP1 and BRCA1 in TNBC. | | | | | | |
| --- | --- | --- | --- | --- | --- | --- |
| Variables | Number of patients |  | NuSAP1 expression | |  | *P* value |
|  |  |  | Negative n (%) | Positive n (%) |  |  |
| BRCA1(IHC) |  |  |  |  |  |  |
| Negative | 107 |  | 59(39.3) | 48(32.0) |  | 0.484 |
| Positive | 43 |  | 21(14.0) | 22(14.7) |  |  |

**Abbreviations**: BRCA1, breast cancer type 1 susceptibility protein; NuSAP1, Nucleolar spindle-associated protein. *P*^a^ value was calculated using Pearson's χ.
