# Supplementary material for: High Levels of Nucleolar Spindle-Associated Protein and Reduced Levels of BRCA1 Expression Predict Poor Prognosis in Triple-Negative Breast Cancer
Source: PLoS One. 2015 Oct 20;10(10):e0140572. doi: 10.1371/journal.pone.0140572 (PMC4618922; doi:10.1371/journal.pone.0140572)
Supplement: S2 Table — (DOCX) [file pone.0140572.s004.docx]

| **Table S2** Correlation between clinicopathologic variables and expression of BRCA1 in the subgroup of TNBC. | | | | | | |
| --- | --- | --- | --- | --- | --- | --- |
| Variables | Number of patients |  | BRCA1 expression | |  | *P*^a^ value |
|  |  |  | Negative n (%) | Positive n (%) |  |  |
| Total | 150 |  | 107(71.3) | 43(28.7) |  |  |
| Age |  |  |  |  |  | 0.314 |
| ≤50 years | 76 |  | 57(38.0) | 19(12.7) |  |  |
| ＞50years | 74 |  | 50(33.3) | 24(16.0) |  |  |
| Menopausal status |  |  |  |  |  | 0.857 |
| Premenopause | 75 |  | 53(35.3) | 22(14.7) |  |  |
| Postmenopause | 75 |  | 54(36.0) | 21(14.0) |  |  |
| Tumor size |  |  |  |  |  | 0.794 |
| ≤2cm | 71 |  | 50(33.3) | 21(14.0) |  |  |
| >2, ≤5cm | 71 |  | 52(34.7) | 19(12.7) |  |  |
| >5cm | 8 |  | 5(3.3) | 3(2.0) |  |  |
| Lymph node status |  |  |  |  |  | 0.106 |
| Negative | 93 |  | 62(41.3) | 31(20.7) |  |  |
| Positive | 57 |  | 45(30.0) | 12(8.0) |  |  |
| Grade |  |  |  |  |  | 0.168 |
| 1 or 2 | 99 |  | 67(44.7) | 32(21.3) |  |  |
| 3 | 51 |  | 40(26.7) | 11(7.3) |  |  |
| TNM |  |  |  |  |  | 0.676 |
| I | 49 |  | 37(24.7) | 12(8.0) |  |  |
| II | 79 |  | 54(36.0) | 25(16.7) |  |  |
| III | 22 |  | 16(10.7) | 6(4.0) |  |  |
| **Abbreviations**: BRCA1, breast cancer type 1 susceptibility protein. *P*^a^ value was calculated using Pearson's χ. | | | | | | |
